# Risk of dementia following herpes zoster infection among patients undertreatment versus those not: A systematic review and meta‐analysis

**DOI:** 10.1002/hsr2.1941

**Published:** 2024-03-13

**Authors:** Sangharsha Thapa, Sangam Shah, Abhinav Bhattarai, Mohammad Yazdan Panah, Swati Chand, Omid Mirmosayyeb

**Affiliations:** ^1^ Westchester Medical Center New York Medical College Valhalla NY USA; ^2^ Tribhuvan University Institute of Medicine Maharjgunj Nepal; ^3^ Students Research Committee Shahrekord University of Medical Sciences Shahrekord Iran; ^4^ Isfahan Neurosciences Research Center Isfahan University of Medical Sciences Isfahan Iran

**Keywords:** antiviral, dementia, herpes zoster, treatment, varicella–zoster virus

## Abstract

**Background and Aims:**

According to the previous studies, herpes zoster (HZ) has been associated with cognitive function and dementia. There is a hypothesis claiming that dementia risk may be reduced by receiving the antiviral treatment for HZ. The purpose of this systematic review and meta‐analysis was to shed light on the association between dementia and HZ in individuals receiving and not receiving antiviral medications.

**Methods:**

Studies investigating the association between HZ and dementia were identified through a systematic search in PubMed/MEDLINE, Scopus, Embase, Google Scholar, and Cochrane Library databases from January, 2000 to April, 2022. Data on the risk of dementia in HZ‐infected patients under and not under antiviral treatment were extracted. The meta‐analysis was conducted using a random‐effects model. The modified ROBIN‐I tool was used to evaluate the risk of bias assessment. By utilizing the funnel plots, publication bias was investigated.

**Results:**

Six cohort studies on 538,531 patients were included. The overall risk of bias assessment was moderate. According to evidence‐based cohort studies, there was a significant direct association between HZ and risk of dementia in patients with HZ, who did not receive antiviral treatments (hazard ratio [HR]: 1.15, 95% confidence interval [CI]: 1.03 to 1.28, *p* = 0.01). On the other hand, there was an inverse relationship between HZ and risk of dementia among patients with HZ, who received antiviral treatments (HR: 0.68, 95% CI: 0.59 to 0.77, *p* < 0.001).

**Conclusions:**

This study demonstrated that antiviral therapies may significantly lower the risk of dementia in patients with HZ. This study also confirmed that patients with HZ, without receiving antiviral therapies, may have an increased risk of developing dementia. Further longitudinal research is warranted in this area.

## 1 INTRODUCTION

Dementia is a syndrome caused by various factors.^1^ The symptoms of dementia include memory impairments, learning difficulties, executive dysfunction, perceptual‐motor impairments, difficulty paying attention, and problems with language expression.^2^ These cognition deficits should be severe enough to hamper daily function and activities. Multiple neuropathologic processes may cause dementia and share common pathophysiological mechanisms like hypoxia, oxidative stress, neuroinflammation, blood–brain permeability, and neurodegeneration.^3^ Dementia has recently emerged as a serious public health concern on a global scale.^4^ Dementia prevalence is rising at the fastest rate, especially in the aging population. As the population ages and dementia cases increase by approximately 7.7 million annually, dementia is now a major social burden.^5^ Over 50 million people worldwide suffer from dementia; one new case arises every 3 s.^6^ Much research has been conducted recently on the relationship between the infectious agent and dementia. A temporal relationship has been found between infectious diseases and dementia in some studies. Among various infectious agents, herpes zoster (HZ) may have significant associations with dementia; however, this association is controversial.^7^, ^8^

Latent viral infections may increase the risk of dementia. One of these infections is HZ, a disease caused by varicella‐zoster virus (VZV).^2^, ^9^ The VZV is a neurotrophic virus that lies dormant in the sensory ganglia. When VZV reactivates after chickenpox, it travels along the axons of sensory nerves, causing HZ.^9^ The reactivation of the virus can cause neuroinflammation, which can result in the formation of misfolded oligomers and the accumulation of amyloid plaques and neurofibrillary tangles made up of hyperphosphorylated tau protein.^10^, ^11^, ^12^ VZV and herpes simplex virus type 1 (HSV1) are herpesviruses implicated in Alzheimer's disease (AD).^13^ Dementia is most commonly caused by AD. Patients with AD have observed marked neuroinflammation in their brains.^14^

According to previous studies, it has been shown that there was a significant association between HZ and the risk of dementia,^2^, ^8^, ^15^ while some of them found opposite findings.^16^, ^17^ A meta‐analysis focused on non‐randomized observational studies determined no significant relationship between HZ and dementia.^18^ HZ can lead to cerebral vasculopathy and stroke, particularly if the cranial nerves are affected, resulting in neurologic damage.^13^ Previous studies found that the incidence of stroke and vasculopathy was higher in patients with herpes zoster ophthalmicus (HZO) than in other types of HZ.^19^, ^20^, ^21^, ^22^ Dementia may also be protected from infection by antiviral treatments against these viruses.^15^

Antiviral drugs reduce acute pain and zoster severity, speed up convalescence, and decrease post‐herpetic neuralgia.^23^ It is commonly used to treat HZ with acyclovir and valacyclovir.^15^ Previous studies have reported that antiviral treatment may reduce the risk of dementia following HZ.^2^, ^8^ Longer durations of antiviral therapy were associated with greater protective effects on dementia incidence than shorter durations.^24^

HZ may be regarded as a potentially modifiable risk factor for dementia.^25^ Furthermore, several cohort studies found that herpes simplex virus (HSV) infection treatment with anti‐herpetic drugs decreased the risk of dementia.^2^, ^8^, ^15^ Through a systematic review and meta‐analysis, this study was designed to gain a clearer understanding of the role of HZ in the risk of dementia and the role of antiviral treatment in preventing dementia following HZ among cohort studies.

## 2 METHODS

### 2.1 Search strategy and study selection

**Figure 1:**
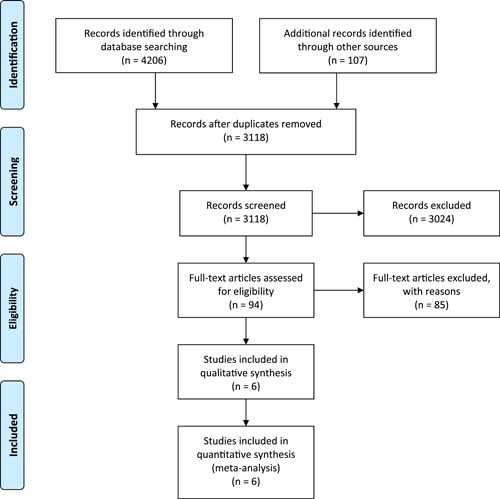
Flow diagram summarizing the criteria for selecting eligible studies under PRISMA. PRISMA, Preferred Reporting Items for Systematic Reviews and Meta‐Analyses.

PubMed/MEDLINE, Scopus, Embase, Google Scholar, and Cochrane Library were systematically searched for all relevant literature published between January 2000 and April 2022, using the syntax (“Dementia” OR “Dementias” OR “Senile Paranoid Dementia” OR “Senile Paranoid Dementias” OR “Familial Dementia” OR “Familial Dementias”) AND (“Herpes Zoster” OR “Zona” OR “Shingles” OR “Varicella Zoster” OR “VZV” OR “Chickenpox”). This study adhered to the Preferred Reporting Items for Systematic Reviews and Meta‐Analyses (PRISMA) guidelines (Figure 1).

### 2.2 Eligibility criteria

All studies possessing the following features were included in the review:

1. The comparative‐cohort studies.
2. The sample size of HZ‐infected patients was at least 500.
3. Studies consider the incidence of dementia in patients with HZ.
4. Patients with HZ were compared for incidence of dementia between those receiving antiviral treatment and those receiving no treatment.

The study excluded case reports, editorials, review articles, and articles whose full text was unavailable in English.

### 2.3 Study selection

Initially, two reviewer (SS and AB) independently screened the titles and abstracts of the search results in the EndNote software version X9 to determine the relevant articles. After that, the reviewers evaluated the full text to identify the final included articles. Additionally, references of previous reviews and included studies were manually screened to discover the further studies. Disagreements that arose during the selection of studies were resolved through discussion.

### 2.4 Data extraction and risk of bias assessment

To extract data from included studies, a predetermined data extraction sheet was designed that included (1) the name of the first author of the study, (2) the country of study, (3) the number of infected patients with HZ, (4) age, (5) gender, and (6) study findings. Two authors (ST and MYP) extracted these data from included studies.

Two authors (ST and OM) used a modified ROBINS‐I tool^26^ to assess the risk of bias in these studies. There are six bias domains in the tool assessment: (1) selection of comparison groups, (2) bias due to confounding, (3) ascertainment of exposure, (4) measurement of outcomes, (5) missing data, and (6) reporting of results. Depending on the type of bias, a domain could have low risk if all the domains had low risk, moderate if any of the domains had a moderate risk, serious if any of the domains had a serious risk, and critical if any domain had a critical risk.

### 2.5 Statistical analysis

Using Review Manager 5.4 by Cochrane Collaboration, the author (SS) performed the meta‐analysis on the extracted data.^23^ *I* ^2^ statistics were used to determine how heterogeneous the studies were, with values more than 50% considered high heterogeneity. An *I* ^2^ statistic was used to determine whether a fixed‐effects or random‐effects model should be applied based on the extent of heterogeneity. The variance of the distribution of true effect sizes, “tau^2^,” was estimated using DerSimonian and Laird method. The pooled hazard ratio (HR) was utilized to assess the risk of dementia in patients with HZ. Using the general inverse‐variance approach, the pooled HR was determined with a 95% confidence interval. A forest plot was created to interpret our findings. Furthermore, publication bias was assessed using a funnel plot. To examine the impact of larger studies on the pooled effect, sensitivity analyses were conducted by deleting one study at a time during the analysis. All tests were considered statistically significant at less than 0.05.

## 3 RESULTS

### 3.1 Search results and study selection

A total of 4206 studies were retrieved from the literature search. Following the removal of duplicates, 3118 studies were screened for the title and abstract. Subsequently, 94 studies were forwarded for full‐text investigation. The full‐text evaluation resulted in six studies being included. In the PRISMA diagram, there is a detailed explanation of the selection process for studies (Figure 1).

### 3.2 Risk of bias among the studies

**Figure 2:**
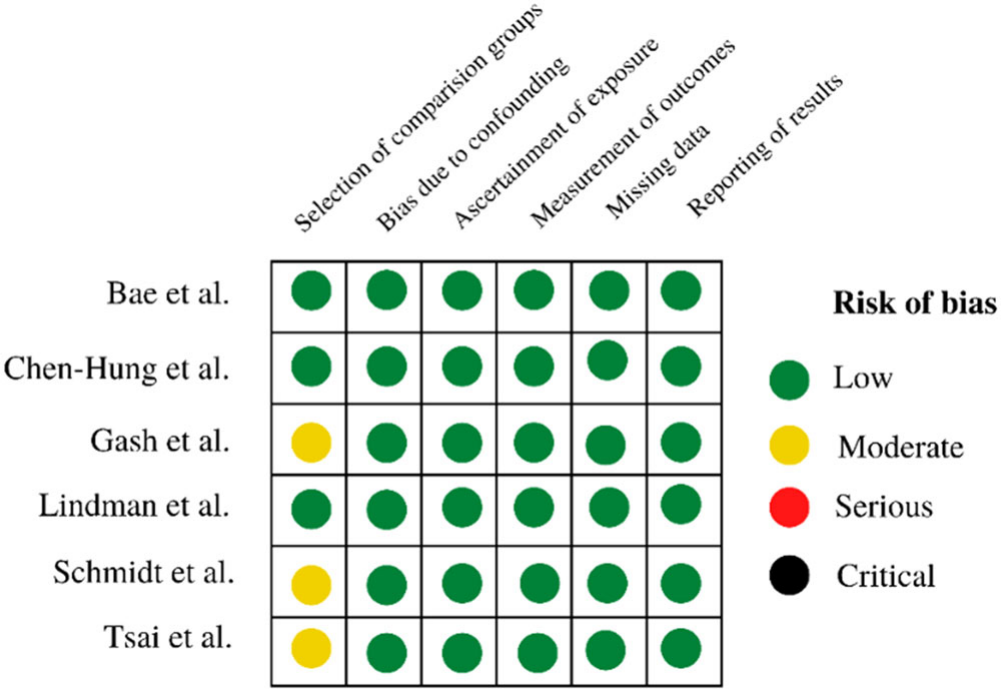
Risk of bias summary of the included studies. A moderate risk of bias indicates that the study is sound as a non‐randomized study in relation to this domain but cannot be compared with a well‐conducted randomized trial.

This systematic review and meta‐analysis included six studies. Three studies^18^, ^19^, ^24^ were deemed low risk of bias, while three^20^, ^25^, ^26^ exhibited a moderate overall risk. Figure 2 presents the results of the risk of bias assessment. No research reported a significant and detrimental risk of bias.

### 3.3 Descriptive characteristics of the included studies

Six studies with 538,531 participants met the study's eligibility criteria and were analyzed qualitatively and quantitatively. All included studies were published between the years 2017 and 2022. Three studies were from Asia, and three studies were from Europe. The least number of HZ‐infected patients was present in the study by Tsai et al. In all studies, patients over 60 years of age were included. The distribution of male and female participants in the studies varied. While two research revealed insignificant results, four studies found a significantly higher risk of dementia in patients with HZ. Three studies investigated the risk of dementia in patients with HZ receiving antiviral treatment. These studies indicated a significant decrease in the risk of dementia in those receiving antiviral treatments. Table 1 displays the descriptive attributes of the included studies.

**Table 1:** Descriptive characteristics of the included studies.

Author, Years	Country	No. of patients with herpes zoster infection	Age (mean ± SD)	Gender (female: %; male: %)	Key findings
Gash et al., 2022^16^	United Kingdom	177,144	>60	32%; 68%	Herpes zoster was not associated with an increased risk of dementia.
Schmidt et al., 2022^27^	Denmark	247,305	64	41%; 59%	The association between herpes zoster and dementia was insignificant, except for patients with pre‐existing central nervous system disease.
Lindman et al., 2021^15^	Sweden	39,526	73.5 ± 10.5	60%; 40%	Compared to the untreated group, antiviral‐treated patients with herpes zoster experienced a significantly lower incidence of dementia.
Bae et al., 2020^8^	South Korea	34,505	61 ± 9.4	55%; 45%	Herpes zoster is associated significantly with dementia in untreated patients (*p* < 0.01). The incidence of dementia was significantly lower in antiviral‐treated patients (*p* < 0.01).
Chen Hung et al., 2018^2^	Taiwan	39,205	>60	NR	Antiviral treatment significantly reduced the risk of dementia in patients with herpes zoster.
Tsai et al., 2017^19^	Taiwan	846	62.2 ± 12.5	50%; 50%	Patients with active or past herpes zoster infection have a significantly higher risk of developing dementia.

Abbreviation: NR, not reported.

### 3.4 Association between herpes zoster and risk of dementia in patients who did not receive antiviral treatments

**Figure 3:**
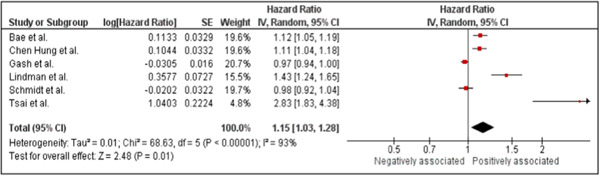
Forest plot showing the association between herpes zoster infections and dementia among patients with herpes zoster without receiving antiviral treatments.

A meta‐analysis of six studies was conducted on the association of HZ with dementia in patients without antiviral treatments. Considering the data's high heterogeneity (*I* ^2^ = 93%), the pooled HR was derived from a random‐effect model. The forest plot (Figure 3) showed a positive association between HZ and dementia (HR: 1.15, 95% CI: 1.03 to 1.28, *p* = 0.01). Thus, according to the statistics, patients with HZ and without antiviral treatments have an increased risk of developing dementia.

The sensitivity analysis showed a fluctuation of the significant findings to insignificant when two studies^15^, ^19^ were removed during the analysis.

**Figure 4:**
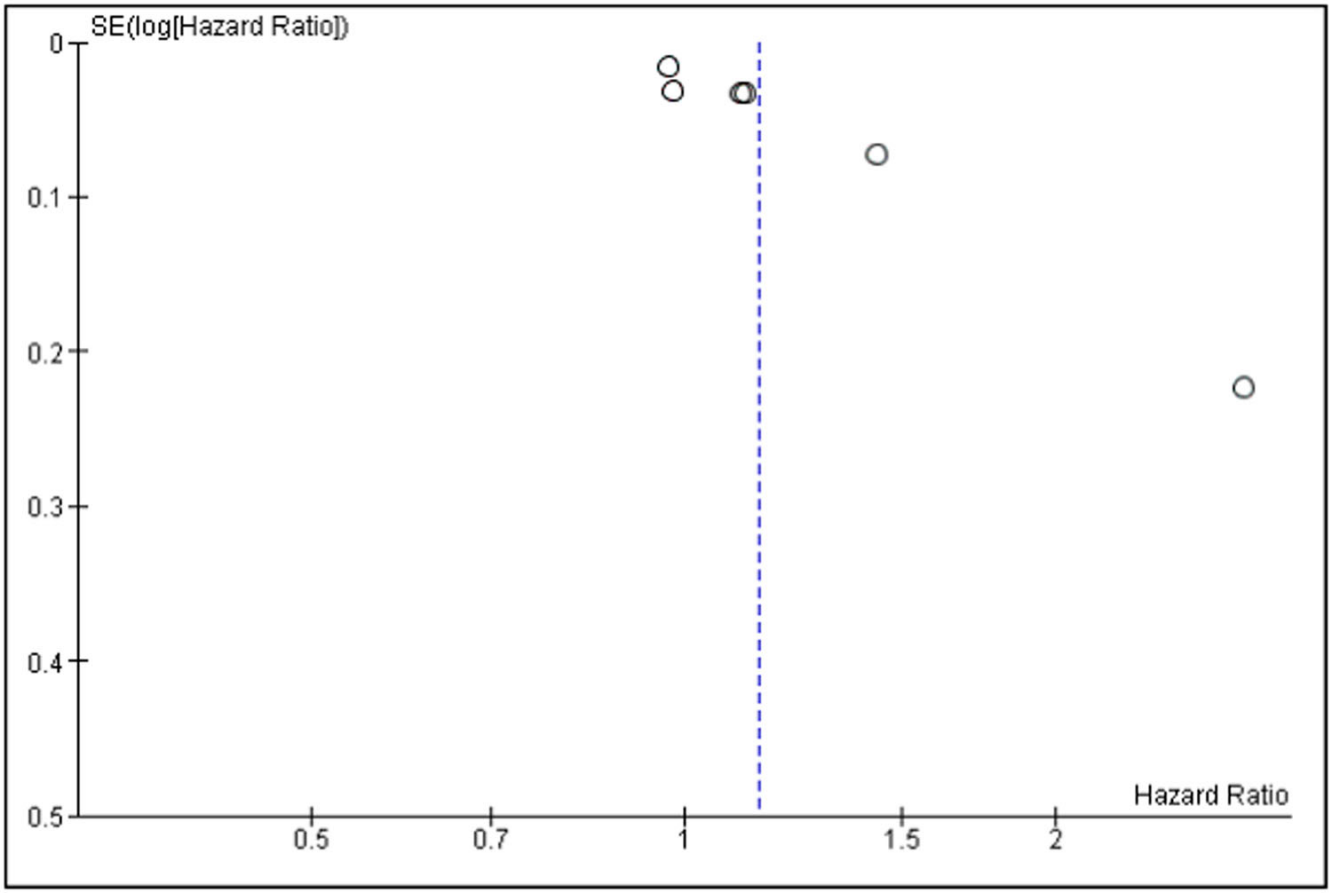
Funnel plot of studies analyzing the association between herpes zoster infection and dementia in patients with herpes zoster without receiving antiviral treatment.

Figure 4 illustrates the funnel plot analysis for evaluating the publication bias. Considering the asymmetrical shape of the funnel plot, potential publication bias may have affected the findings of this meta‐analysis.

### 3.5 Association of herpes zoster with dementia in patients who received antiviral treatment

**Figure 5:**
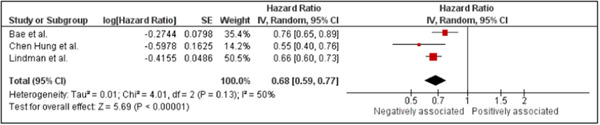
Forest plot showing the association of herpes zoster infections with dementia among patients with herpes zoster receiving antiviral treatments.

The meta‐analysis included three studies on the association of HZ with dementia in patients with antiviral treatments. Considering the high heterogeneity (*I* ^2^ = 50%) within the data, the pooled HR was derived using random‐effects models. The forest plot (Figure 5) indicated an inverse relationship between HZ and dementia in patients receiving antiviral treatments (HR: 0.68, 95% CI: 0.59 to 0.77, *p* < 0.001). Consequently, patients with HZ under antiviral treatments have a decreased risk of developing dementia.

In the sensitivity analysis, significant findings fluctuated from significant to insignificant following the removal of one study.^8^

## 4 DISCUSSION

This systematic review and meta‐analysis showed that recent HZ may be associated with an increased risk of dementia. As a result of this study, it has been established that the HZ and the onset of dementia are related. Additionally, the meta‐analysis showed that antiviral medications reduce the dementia risk associated with HZ. Our findings and results focus on the potentially protective nature or effect of systemic antiviral medications in patients with first‐episode HZ on dementia and provide crucial information on the relationship between HZ and risk of dementia.

In the present study, five studies^2^, ^8^, ^15^, ^19^, ^27^ from various parts of the world demonstrated that dementia and HZ had a significant association when comparing them to the general population without HZ. Four studies showed an increased risk of dementia among patients with HZ.^2^, ^8^, ^15^, ^19^ Overall, our substantial meta‐analysis established that HZ patients with no antiviral treatment were at a higher risk of dementia than those who received antiviral therapy. Contrary to our findings, two studies^16^, ^17^ and a meta‐analysis^18^ found no substantial association between HZ and the risk of developing dementia. Previous meta‐analysis^18^ employed an odds ratio with fewer included studies than ours. Still, we took a different approach by prioritizing cohort studies with a larger sample size to investigate the HR of dementia in patients with HZ. Additionally, we employed adjusted HR to mitigate the impact of covariates to the greatest extent possible. This approach may enhance the reliability of our findings. In a systematic review and meta‐analysis, it was found that VZV reactivation from clinically diagnosed cases (specifically ophthalmic zoster) increased the risk of dementia, whereas brain or serum samples of VZV infection or reactivation were not associated with dementia.^7^ The HZ that affects the trigeminal nerve's ophthalmic branch (ophthalmic HZ) increases the risk of dementia to 2.97 (95% CI: 1.89–4.66).^19^ Unlike our finding, a case‐control study found that Individuals of either sex or age are not at an increased risk of developing neurodegenerative dementia due to HZ.^17^

Multiple biological pathways can explain the virus‐related sequelae of dementia. Bubak et al. found that patients with HZ had significantly higher plasma amyloid levels than non‐zoster controls. It is possible that the plasma of infected patients with zoster may contain factors that contribute to the aggregation of amyloidogenic peptides, explaining the accelerated disease progression following zoster.^28^ The VZV can be found in the brain and induces amyloid‐beta and tau, which exhibit the hallmark pathological characteristics of AD. There is evidence that specific causes of senile dementia may be linked to HZ.^29^ It has been proposed that the insulin‐degrading enzyme (IDE) plays a significant role in the pathophysiology of HZ dementia. IDE degrades several small proteins, including amyloid beta.^30^ Evidence shows that IDE is a VZV receptor mediating infection and cell‐to‐cell transmission.^31^

VZV might also cause dementia through other mechanisms. The reactivation of the neurotrophic VZV can lead to a cascade of neuroinflammation, glial activation, cerebral vasculopathy, or direct neural damage that can contribute to the development of dementia or cognitive dysfunction.^13^, ^32^ Direct virus invasion into the brain may be responsible for the association between HZ and dementia. However, CNS symptoms may not be present at the time of presentation.^33^ Additionally, VZV‐induced vasculopathy could also explain associations between HZ and dementia.^2^ In patients with HZO, dementia may be attributed to cerebral vasculopathy, as the virus can reside in the cerebral arteries of these individuals.^33^, ^34^ Wang et al. found that VZV leads to systemic inflammation and the production of inflammatory cytokines, including tumor necrosis factor (TNF‐α) and interleukin‐6 (IL‐6).^34^ Studies have shown that levels of inflammatory markers, such as TNF‐α, IL‐6, and C‐reactive protein, elevate before the manifestation of clinical symptoms associated with various types of dementia, including AD and vascular dementia.^35^, ^36^ There are limited data and studies to support the theory of the pathophysiology of HZ to cause dementia. Nonetheless, these pathways remain considered the most significant pathophysiological factors underlying HZ‐induced dementia.

This study found that patients with HZ were less likely to develop dementia when they took anti‐herpetic medications. Based on the results of the meta‐analysis, HZ is negatively associated with dementia in patients who are taking antiviral medication (HR: 0.76; 95% CI: 0.61–0.94; *p* = 0.01). Three studies^2^, ^8^, ^15^ demonstrated that anti‐herpetic treatment could reduce the risk of dementia. Antiviral medications have been shown to reduce acute pain and the severity of zoster, accelerate the healing process, and alleviate post‐herpetic neuralgia.^23^ As a result of reducing inflammation, antiviral drugs may potentially mitigate the adverse effects associated with post‐zoster.^37^ According to a large case‐series study, patients who received oral antiviral therapy for HZ were at a lower risk of stroke than those who did not receive treatment.^38^ In previous research, MRI results after antiviral treatment of VZV intracranial vasculopathy showed thickened vessel walls and decreased enhancement.^2^, ^39^ A Taiwanese study found that the incidence of dementia was approximately half that of patients who were not treated with antiviral medication.^2^ According to another survey, roughly a quarter of the patients in the treated group suffered from post‐zoster dementia compared to the untreated group.^8^ It may be due to the rigorous adjustment of possible confounders that antiviral therapy has relatively weak effects on reducing dementia risk in some studies. A study found that patients with HZ treated in Denmark had a 10% lower risk of dementia diagnosis, while those diagnosed and treated for HZ in Germany did not experience a difference in diagnosis.^40^ Additionally, the link between VZV vaccination and reduced risk of dementia has been demonstrated in previous studies.^41^, ^42^ It is possible that differences in the findings of studies can be attributed to differences in the methods used. The adjustment of covariates also contributes significantly to the findings of studies.

### 4.1 Strengths and limitations

The results of this systematic review and meta‐analysis are the first to demonstrate the importance of using antiviral agents early on during HZ to reduce the risk of dementia. The risks of dementia following HZ were investigated in two systematic reviews and meta‐analyses^7^, ^18^; however, a few studies were included, and the role of antiviral drugs in reducing the risk of dementia has not been examined. Our thorough search approach, which included publications from seven medical databases and gray literature, is one of our review's most vital points. Including extensive cohort studies strengthens the results of this study (at least 500 individuals per research). In specific investigations, Nucleic acid of Herpesvirus (VZV) has been found in post‐mortem brains. However, whether this is due to reactivation or to peri‐mortem alterations related to neurodegeneration is still unclear. We excluded these studies from this meta‐analysis to avoid confusing and ambiguous results.

The present study has some limitations. HZO, as opposed to other HZ, has been observed to enhance the incidence of dementia in population‐based cohorts. It has been hypothesized that compared to HZ, which affects more peripheral areas, the virus is more likely to infiltrate the CNS in cases of HZO. To demonstrate the connection between VZV and dementia, our meta‐analysis included both HZ and HZO; however, due to a dearth of comparable data, we could not demonstrate the relationship independently. Further research is needed to differentiate between HZ‐associated dementia without and with ophthalmics. This research will reveal whether patients with HZ with ocular problems might be more likely to get dementia. This study could not determine if dementia is linked to recent, old, or reactivated HZ. There was no consideration of different antiviral treatment regimens in this study, and it is also possible that patients who required antiviral treatment may have more comorbidities and be sicker than patients who did not receive antiviral treatment or required antiviral treatment, so more research is needed to determine this concern. As a whole, this study identified a lack of longitudinal studies with repeated exposure measurements, a lack of confounding controls, including age and sex, as well as a large number of small studies that could not identify an effect. Regarding these limitations, the findings of this study should be cautiously interpreted.

## 5 CONCLUSION

It was concluded that HZ may contribute significantly to the development of dementia among patients who are not receiving antiviral therapies. Furthermore, it demonstrated that antiviral treatments had the capability of reducing the likelihood of dementia as a result of HZ. Based on the findings of this study and those of earlier studies, it is highly advised to take even more proactive measures in treating herpes reactivation symptoms with antiviral medications to potentially low. Additional longitudinal studies are required to elucidate the impact of antiviral treatments on risk of dementia among patients with HZ.

## AUTHOR CONTRIBUTIONS

**Sangharsha Thapa**: Conceptualization; methodology; writing—review and editing. **Sangam Shah**: Data curation; investigation; validation; visualization. **Abhinav Bhattarai**: Data curation; formal analysis; software. **Mohammad Yazdan Panah**: Data curation; investigation; writing—original draft; writing—review and editing. **Swati Chand**: Supervision; writing—review and editing. **Omid Mirmosayyeb**: Conceptualization; validation; writing—review and editing.

## CONFLICT OF INTEREST STATEMENT

The authors declare no conflict of interest.

## TRANSPARENCY STATEMENT

The corresponding author affirms that this manuscript is an honest, accurate, and transparent account of the study being reported; that no important aspects of the study have been omitted; and that any discrepancies from the study as planned (and, if relevant, registered) have been explained.

## ETHICS STATEMENT

The ethical committee approval was not required given the article type.

## Data Availability

All the required information is in the manuscript itself.
